# An empirical study of college students’ reading engagement on academic achievement

**DOI:** 10.3389/fpsyg.2022.1025754

**Published:** 2022-11-10

**Authors:** Xiao-Wu Wang, Yu-Juan Zhu, Yi-Cheng Zhang

**Affiliations:** ^1^Anhui Xinhua University, Hefei, China; ^2^Institute of Higher Education, Anhui Xinhua University, Hefei, China; ^3^Quality Education Research Center, Anhui Xinhua University, Hefei, China; ^4^School of Management, University of Science and Technology of China, Hefei, China

**Keywords:** metaverse, reading engagement, academic achievement, college organizational endowment, college reading management

## Abstract

With the popularity of Internet technology, reading has developed in the direction of digitalization and mobileization. And entering the metaverse era, both the subject and object of reading may be redefined, presenting a new developmental pattern. This process brings a crisis to reading, such as the fragmentation of reading, the obstruction of reading needs, and the replacement of classical reading. However, reading is still an important way for college students to acquire new knowledge, broaden their horizons and improve their skills. The existence of reading crises inevitably affects the academic achievement of college students. Therefore, from the perspective of university management, this paper conducts regression analysis on 1,155 effective samples of colleges and universities in Anhui Province, extracts the factors that affect college students’ reading engagement, and further explores the relationship between college students’ reading engagement and academic achievement. The study concluded that: (1) in terms of family reading culture, students who grow up in families with good family reading culture perform better in reading engagement. The amount of family books, family reading education and family reading atmosphere all have significant positive effects on reading time and reflective reading strategies of college students. (2) In the cultivation of reading habits in colleges and universities, the course-driven mechanism and the atmosphere stimulating mechanism have a significant positive effect on students’ reading time. The course-driven mechanism, resource supporting mechanism and atmosphere stimulating mechanism have a significant positive effect on the critical reading strategy of college students. (3) In terms of reading time, it is only found that the reading time spent on paper books has a significant positive effect on college students’ academic achievement and professional quality. (4) In terms of reading strategies, the replicative reading strategy only has a significant positive effect on the improvement of college students’ academic achievement and professional quality. The critical reading strategy has a significant positive effect on the professional quality, general ability and career planning ability of college students.

## Introduction

The year 2021 is regarded as the “meta-universe year” (Zhao et al., 2021), and the phenomenon of “meta-universe” has received widespread attention, reflecting the new trend of digital technology development after new technologies such as big data, blockchain, 5G and cloud computing. The metaverse is linked and created by using technology, a virtual world mapped and interacted with the real world, and a digital living space with a new social system. Combing through the literature related to metaverse reveals that many scholars focus on the impact of metaverse on games, literary travel, education and other fields, but there are few studies on the connection between metaverse and reading and how metaverse will bring changes to reading. With the popularity of Internet technology, reading has developed in the direction of digitalization and mobilization. And entering the era of metaverse, both the subject and the object of reading may be redefined and take on a new developmental shape. This process brings a crisis to reading, such as reading fragmentation, hindered reading demand, and the replacement of classical reading (Cai and Zhao, 2022; Dwivedi et al., 2022; Zhu, 2022).

The acquisition of intellectual capabilities and development of high level manpower which is the goal of tertiary education implies a crop of students with good study habits because effective study habits and strategies have been attributed to the secret of success in school, graduation, entering the universities as well as the attainment of job advancement (Musingati and Zebron, 2014).

Reading is an important way for contemporary college students to acquire new knowledge, expand their horizons and improve their skills, which is not only beneficial to their academic progress and quality improvement, but also plays an important role in creating a good cultural atmosphere and playing the function of educating people in colleges and universities. As an important means of educating people in colleges and universities, reading is related to the mission of cultivating talents in colleges and universities and the effectiveness of cultivating talents in universities. Therefore, reading for college students is one of the very important research topics in college education management. (Leal-Rodriguez and Albort-Morant, 2019; Gao, 2021; Weli and Nwogu, 2022).

Based on the above, from the perspective of university management, this study explores: (1) What factors affect college students’ reading engagement? (2) What is the influence mechanism of college students’ reading engagement on their academic performance? Thus, the university reading management mechanism can be constructed to improve their academic achievement.

## Literature review and research hypotheses

### College students’ reading engagement

Different scholars hold different views on the connotation of reading engagement, and so far, there is no unified standard. Csikszentmihalyi (1990) believes that reading engagement is a state of concentration. Pearson et al. (2016) believed that reading engagement is the interaction between students’ motivation and strategies. Different scholars have different views on the dimensions of reading engagement. PISA2009 divides reading engagement into personal reading engagement and school reading engagement. Personal reading engagement includes four dimensions: love of reading, diversity of reading, online reading activities and reading time. The school reading engagement specifically includes four dimensions: text interpretation, use of non-consecutive texts, reading activities of traditional literary works, and instrumental text use. Zhang et al. (2014) drew on the PISA2009 definition of reading engagement, and divided reading engagement into reading time, reading quantity and reading interest. Wen et al. (2016) studied reading engagement from three dimensions: length of reading time, amount of reading, and diversity of reading content.

The reading engagement proposed in this study refers to the time and energy input of college students in the process of reading, which specifically includes college students’ reading time and reading strategies (Brookbank et al., 2018; McDaniel, 2018). See Table 1 for details.

**TABLE 1:** Specific indicators of reading engagement.

Reading time	Paper reading time		The median reading time of paper books and electronic books
	Electronic reading time	Book	(1 = less than 0.5, 2 = 0.5 − 1.5, 3 = 1.5 − 2.5, 4 = 2.5 − 3.5, 5 = more than 3.5) (unit: hour)
Reading strategies	Replicati ve strategy	Reading	The theory and method of memorizing reading
			Facts and situations
			Take notes with emphasis
	Criti cal strategies	Reading	Read with questions, analyze and synthesize information or experiences from different perspectives in reading
			Carefully studying and thinking while reading
			Look up supplementary materials, discuss reading problems with others

### Academic achievement

Research on the academic achievement of college students can be traced back to the 1960s. In 1966, the American Educational Advisory Council created the “Cooperative Program on Institutional Research.” After the 1990s, countries all over the world began to pay attention to the investigation and research on the academic achievement of college students. This is not only an effective way to judge the value growth of college students, but also an important way to explore the learning and development of college students, and an effective method to measure the quality of college education (Douglass et al., 2012). Park et al. (2014) summarized the academic achievement of college students as knowledge, values and attitudes, skills or appropriate behaviors. American university scholars developed a standardized test tool CLA (Collegiate Learning Assessment), which reflects the academic achievement status of college students by measuring critical thinking ability, analytical reasoning ability, problem-solving ability and communication ability. Developmental psychology believes that there is an interaction mechanism between value orientation and behavioral choice, and there is a causal relationship between academic achievement orientation and behavior, that is, college students with higher academic achievement will have better future development (Morgan, 2004; Aharony and Bar-Ilan, 2018). Maniaci et al. (2021) evaluated the relationship between healthy lifestyle and academic performance of 373 Italian adolescents, and found that academic achievement was conducive to healthy lifestyle and good eating habits.

### Reading engagement and academic achievement of college students

According to the input-environment-output model proposed by Astin (1984), output refers to students’ ability, that is, the target of education and teaching in colleges and universities, input refers to the personal characteristics of students before receiving higher education, including the student’s family background, academic qualifications before admission, etc., environment refers to all kinds of actual experiences that students experience in universities and colleges, including those from course teaching, campus activities, social practice, and others (Astin, 1984).

A study environment therefore refers to the physical, social and psychological situations that affect a student’s well being as well his studies. A study environment must be safe, healthy and promote the study life of a student (Dempsey, 2020). A satisfying learning environment is conducive to college students’ reading engagement (time and energy), which in turn enhances their academic achievement. Therefore, the more college students participate in various practical activities, the better their comprehensive ability and quality will be improved (Camelo and Elliott, 2019; Wu, 2019; Marley and Wilcox, 2022).

### Research hypothesis

Reading is essentially the inheritance and innovation of knowledge (object) in the interactive relationship between subject (reader) and carrier (reading material). The differences of reading subjects (readers) mainly depend on individual preconditions and post-environments. In the preconditions, family reading characteristics (mainly the pros and cons of the students’ family cultural capital) play a leading role. In the post-endowment environment, the cultivation of reading habits in colleges and universities plays a leading role. Different kinds of colleges and universities play different roles in the cultivation of reading habits, which affects reading engagement. The object of reading is composed of explicit knowledge and tacit knowledge. Explicit knowledge is mainly composed of disciplinary professional knowledge, interdisciplinary integration knowledge and fragmented knowledge, while tacit knowledge is obtained through comprehension, and in university campus, it is mainly manifested as scholarly campus and cultural influence. Reading carriers are mainly composed of paper carriers and digital carriers. Under the background of technological change, intelligent knowledge production (IR, AI), interactive knowledge transmission and lively knowledge experience (AR, VR) are the inevitable trends of the development of reading carriers.

Therefore, this study explores the influence mechanism of college students’ reading engagement on the improvement of academic achievement from the perspective of students and universities. Based on this, this study puts forward the following hypotheses:

**Hypothesis 1:** The characteristics of reading subjects (preconditions and college endowments) have a significant influence on the academic achievement of college students. The better the subject endowment, the better the academic achievement of college students.

**Hypothesis 1a:** Controlling other variables, the better the college students’ pre-existing conditions, the better their academic achievement;

**Hypothesis 1b:** Controlling other variables, the better the endowment of college students in colleges and universities, the better their academic achievement;

**Hypothesis 2:** College students’ reading engagement has a significant influence on academic achievement, that is, the more college students engage in reading, the better their academic achievement.

**Hypothesis 2a:** Controlling other variables, the more time college students devote to reading, the better their academic achievement;

**Hypothesis 2b:** Controlling other variables, the better the reading strategy of college students, the better their academic achievement;

**Hypothesis 3:** The cultivation of reading habits in colleges and universities has a significant impact on the academic achievement of college students by influencing their reading engagement, that is, the better the university’s curriculum driving mechanism, resource support mechanism, atmosphere stimulation mechanism and interaction promotion mechanism are played, the better the academic achievement of college students will be.

The logic of this research is to construct the relationship between reading engagement and academic achievement, and explore the influence mechanism of reading engagement on academic achievement by investigating the respective effects of reading engagement at the student level and the university level. The reading engagement (time and strategy) level of college students, from the individual level, is mainly affected by the individual’s preconditions. From the perspective of group characteristics, it is mainly affected by the endowment of colleges and the cultivation of college reading habits. The combined effect of the individual level and the university level constitutes a research system of college students’ reading engagement on academic achievement.

## Research design

### Questionnaire design and data collection

#### Questionnaire design

First of all, literature was sorted out to collect authoritative classical scales, and some mature scales were used for reference. In this study, the reading engagement questionnaire of college students draws on the questionnaire of “Research on Reading Motivation of College Students” by Chen Xiaoli of Jinan University and the questionnaire of “Chinese Reading Behavior in the Digital Age” by Li Xinxiang of Wuhan University. The college students’ academic achievement questionnaire was based on the “University Quality and Student Development Monitoring Project in Beijing” questionnaire and NSSE-China.

Secondly, through discussion among members of the research team and consultation with authoritative experts in this field, the contents of the questionnaire items and the wording of the questionnaire were modified, and the design content of the questionnaire was initially determined. In the first part, the purpose of the study was explained to the research subjects. The second part defines the background data of this study. The third part is mainly about the impact of college students’ reading engagement on academic achievement, including time reading motivation, family reading culture, college reading habit training, college organizational endowment and so on. The fourth part is the basic information of the research object, including age and other demographic variables.

Finally, a small number of qualified samples were screened, and the proposed questionnaire was used for pre-investigation, and the data of the recovered questionnaire was sorted out. At the same time, the reliability and validity of the questionnaire items were tested, and the inconsistent questions were deleted to form the final draft of the questionnaire.

#### Questionnaire distribution and collection

After the preliminary questionnaire survey, the re-adjustment and modification of the questionnaire were completed to improve reliability and validity, and the final questionnaire of this study was formed.

In this study, cluster sampling method was mainly adopted. This method is to merge the units in the population into several non-intersecting and non-repeating sets, that is, cluster; and then use the cluster as a sampling unit to draw samples.

Considering the representativeness and comprehensiveness of the sample, three undergraduate universities in Anhui Province were selected, one is a university of Project 985, one is a government-run undergraduate university, and one is a private undergraduate university. The student sample covers freshmen to seniors, and the majors cover humanities and social sciences, science and engineering. In the questionnaire survey, we gathered the students together and conducted the survey in the form of answering online questionnaires through smartphones. Finally, a total of 1,155 valid questionnaires were obtained.

### Measurement of variables

This study takes the academic achievement of college students as the dependent variable, which includes three dimensions, namely, professional ability, general competence, and career planning ability. The reading engagement of college students was taken as the explanatory variable, and the individual characteristics of college students, reading motivation, family reading culture, college reading habit training and college organizational endowment were used as control variables. The definitions of main explanatory variables, explained variables and control variables are shown in Table 2.

**TABLE 2:** Measurement of variables.

	Variables	Indicators	Variable definitions
Academic achievement	Professional ability	Professional performance improvement	Compositional variable, 1 = strongly disagree, 2 = disagree, 3 = neither agree nor disagree, 4 = agree, 5 = strongly agree
		Self-learning ability	
	*General ability*	Presentation skills	
		Organization and leadership skills	
	*Career planning ability*	Establish future career direction	
		Form phased development goals	
Reading engagement	Reading time	Paper reading time	Interval level of measure, 1 = less than 0.5, 2 = 0.5 − 1.5, 3 = 1.5 − 2.5, 4 = 2.5 − 3.5, 5 = more than 3.5 (unit: hour)
		E-reading time	
	Reading strategies	Replicative reading strategy	Compositional variable, 1 = strongly disagree, 2 = disagree, 3 = neither agree nor disagree, 4 = agree, 5 = strongly agree
		Critical reading strategies	
College students	Individual characteristics	Gender (male-based)	Categorical variable, 0 = male, 1 = female
		Grade (based on freshman year)	Ordinal measurement, 1 = freshman,2 = sophomore, 3 = junior, 4 = senior
		Birthplace (city-based)	Categorical variable, 0 = urban, 1 = rural
		Subject (based on humanities and social sciences)	Categorical variable, 0 = Humanities and social sciences, 1 = Science and Engineering
	Reading motivation	Pragmatic motivation	Compositional variable, 1 = strongly disagree, 2 = disagree, 3 = neither agree nor disagree, 4 = agree, 5 = strongly agree
		Recreational motivation	
		Developmental motivation	
	Family reading culture	Father’s education	Ordinal measurement, 9 = Junior high school, 12 = senior high school, 12 = secondary vocational, 14 = Junior college, 16 = undergraduate, 19 = graduate.
		Mother’s education	
		Family book collection	Interval level of measure, 5 = less than 50, 8 = 60–100, 13 = 110–150, 18 = 160–200, 20 = more than 200 (Unit: thousand)
		Home reading education	Compositional variable, 1 = strongly disagree, 2 = disagree, 3 = neither agree nor disagree, 4 = agree, 5 = strongly agree
		Family reading atmosphere	
Cultivation of reading habits in colleges and universities	Course driven mechanism	Whether to offer reading instruction courses	Categorical variable, 1 = YES, 0 = NO
		Whether to offer courses related to the interpretation of classic works	
		Is there any required reading list for the major you are studying	
	Resource support mechanism	The amount of paper books can meet the reading demand	Compositional variable, 1 = strongly disagree, 2 = disagree, 3 = neither agree nor disagree, 4 = agree, 5 = strongly agree
		E-books are rich in resources and easy to obtain	
		Mobile library, reading client, and online learning platform are easy to use	
		The library enriches book resources according to the needs of students	
	Atmosphere incentive mechanism	Does the school regularly hold large-scale reading activities such as “Reading Festival” and “Reader Month”	Categorical variable, 1 = YES, 0 = NO
		Whether the department or class regularly conducts reading-themed activities	
		In addition to the library, does the school have other reading places such as “Book Bar” and “Reading Corner”	
		The school has many places for reading	
		Various reading activities in school can stimulate my interest in reading	
		After entering school, I became more interested in reading	Compositional variable, 1 = strongly disagree, 2 = disagree, 3 = neither agree nor disagree, 4 = agree, 5 = strongly agree
	Interactive promotion mechanism	Does the instructor of the major course have a recommended reading list	Categorical variable, 1 = YES, 0 = NO
		All my classmates around me love reading	Compositional variable, 1 = strongly disagree, 2 = disagree, 3 = neither agree nor disagree, 4 = agree, 5 = strongly agree
Organizational endowment	Type of university (based on local private undergraduate university)		Categorical variable, 1 = local private university, 2 = government-run undergraduate university, 3 = university of Project 985

### Modeling

In order to verify the mechanism of college students’ reading engagement on college students’ academic achievement proposed in this paper, the following multiple regression models were used to analyze from the student level and the university level. The specific model is as follows:


(1) $${\text{X}}_{i\,j}=\beta_{0}+\beta_{1}\,{\text{A}}_{\text{ij}}+\beta_{2}\,B_{\text{ij}}+\beta_{3}\,C_{\text{ij}}+\beta_{4}\,D_{\text{ij}}+\beta_{5}\,E_{\text{ij}}+\varepsilon_{\text{i}}$$

(2) $${\text{Y}}_{i\,j}=\beta_{0}+\beta_{1}\,{\text{X}}_{\text{ij}}+\beta_{2}\,A_{\text{ij}}+\beta_{3}\,B_{\text{ij}}+\beta_{4}\,C_{\text{ij}}+\beta_{5}\,D_{\text{ij}}+\beta_{6}\,E_{\text{ij}}+\varepsilon_{\text{i}}$$

Among them, X*ij* represents the reading engagement of college students, A*ij* represents the individual characteristics of college students; B*ij* represents the reading motivation of college students; C*ij* represents the reading culture of college students’ families; D*ij* represents the cultivation of reading habits in colleges and universities; E*ij* stands for organizational endowment of colleges and universities; Y*ij* is for academic achievement of college students. β*i* is the regression coefficient.

In Model 1, the dependent variable is X*ij*, and other variables are independent variables, which are used to explore the factors affecting college students’ reading engagement. In Model 2, the dependent variable is Y*ij*, and other variables are independent variables, which are used to explore the impact of college students’ reading engagement on their academic achievement.

## Analysis of empirical results

### Descriptive statistical analysis

In terms of the grade distribution of the sample, there are fewer seniors. The main reason is that the courses of seniors have finished, and many students have been practicing abroad, so the number of seniors in school is small. The sample number of freshmen, sophomores and juniors was relatively balanced, with 238 freshmen, accounting for 20.6%, 436 sophomore students, accounting for 37.7%, 359 juniors, accounting for 31.1 percent. In terms of the types of colleges and universities, 203 students are from 985 colleges and universities, accounting for 17.6%. The number of students from local public undergraduate universities is 283, accounting for 24.5%; the number of local private undergraduate students is 669, accounting for 57.9%. In terms of gender, there are 633 boys, accounting for 54.8%, and 522 girls, accounting for 45.2%. In terms of majors, 382 students are in humanities and social sciences, accounting for 28.5%, while 826 in science and engineering, accounting for 71.5%. In terms of the source of students, 606 students are from rural areas, accounting for 52.5%, and 549 from urban areas, accounting for 47.5%. In terms of the types of high schools, 788 students, accounting for 68.2%, are enrolled in key high schools, and 367 students, or 31.8 percent, are enrolled in regular high schools. From the perspective of annual family income, 58.4% (674 students) have an annual family income of 50,000 yuan or less, 25% (289 students) 60,000–100,000 yuan, and the remaining 17.6% (242 students) 100,000 yuan or more. See Table 3 for details.

**TABLE 3:** Descriptive statistics of the sample.

Variable name	Class	Number	Proportion
Grade	Freshman	238	0.206
	Sophomore	436	0.377
	Junior	359	0.311
	Senior	122	0.106
Types of universities	985 Universities	203	17.60%
	Local public undergraduate universities	283	24.50%
	Local private undergraduate universities	669	57.90%
Gender	Male	633	54.80%
	Female	522	45.20%
Disciplines	Humanities and social sciences	382	28.50%
	Science and engineering	826	71.50%
Origin of students	Village	606	52.50%
	City	549	47.50%
Types of high school	Key high school	788	68.20%
	Normal high school	367	31.80%
Annual household income	50,000 yuan or less	674	58.40%
	600,00–100,000 yuan	289	25.00%
	More than 100,000 yuan	242	17.60%

### Reliability analysis

The reliability test of the questionnaire is mainly to ensure the reliability of the questionnaire, so we conducted the reliability test of the questionnaire by using SPSS21.0. According to the structure of the questionnaire, we selected the following dimensions for reliability analysis, as shown in Table 4.

**TABLE 4:** Reliability analysis.

Variable name	Item	Index	Cronbach’s α coefficient
Personal feature	a1	Gender (based on male)	0.822
	a2	Grade (based on freshman year)	
	a3	Origin of students (based on city)	
	a4	Disciplines (Humanities and Social sciences as the benchmark)	
Reading motivation	b1	Motivation for practicality	0.84
	b2	Motivation for entertainment	
	b3	Motivation of development	
Family reading culture	c1	Father’s education level	0.891
	c2	Mother’s education level	
	c3	Home book collection	
	c4	Family reading education	
	c5	Family reading atmosphere	
Course driven mechanism	d1	Whether to offer reading instruction courses	0.905
	d2	Is there a course on interpretation of the classics	
	d3	Is there a specific required reading list for your major	
Resource support mechanism	e1	The amount of paper books can meet the reading demand	0.919
	e2	Electronic books are abundant and easy to obtain	
	e3	Mobile library, reading client, online learning and other platforms are easy to use	
	e4	The library is rich in book resources according to students’ needs	
Atmosphere excitation mechanism	f1	Whether the school regularly holds “reading Festival,” “Reader Month” and other large-scale reading activities	0.841
	f2	Does the department or class regularly carry out reading themed activities	
	f3	Besides the library, does the school have other reading places such as book bar and reading corner	
	f4	There are many good places to read in school	
	f5	All kinds of reading activities in school can stimulate my interest in reading	
	f6	After entering the school, I became fonder of reading	
Interactive facilitation mechanism	g1	Does the instructor of the major course have a recommended reading list	0.936
	g2	All my classmates around me love reading	
Organizational endowment of universities	h1	Types of universities (based on local private undergraduate universities)	0.912

Cronbach’s α coefficient is used to measure the reliability of the internal consistency of the questionnaire. If the Cronbach’s α coefficient is larger, it indicates that the degree of internal consistency of the questionnaire is higher, and the reliability of the questionnaire results is stronger. It is generally believed that the reliability coefficient should be between 0 and 1. If the reliability coefficient of the questionnaire is above 0.9, it means that the reliability of the questionnaire is very good. If the reliability coefficient of the questionnaire is between 0.8 and 0.9, it indicates that the reliability of the questionnaire is acceptable. If the reliability coefficient of the questionnaire is between 0.7 and 0.8, it indicates that some items of the questionnaire need to be revised. If the reliability coefficient of the questionnaire is below 0.7, it indicates that some items in the questionnaire need to be deleted.

The reliability of this questionnaire can be found from the above table, the reliability of each dimension of the questionnaire is above 0.8, indicating that the reliability of the questionnaire in this paper is good.

### Validity analysis

**FIGURE 1:**
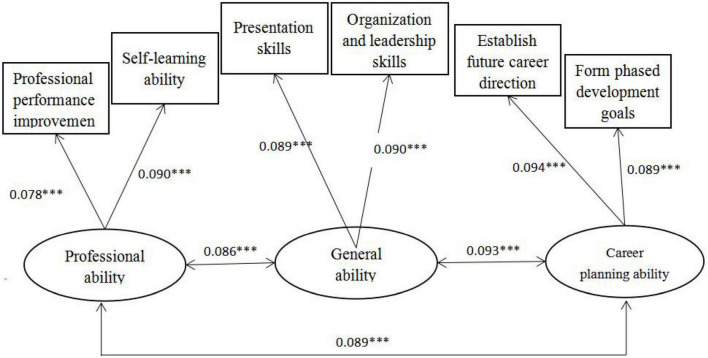
Construct validity analysis chart of academic achievement (after normalization). Significance level *P < 0.1, **P < 0.05, and ***P < 0.01.

**FIGURE 2:**
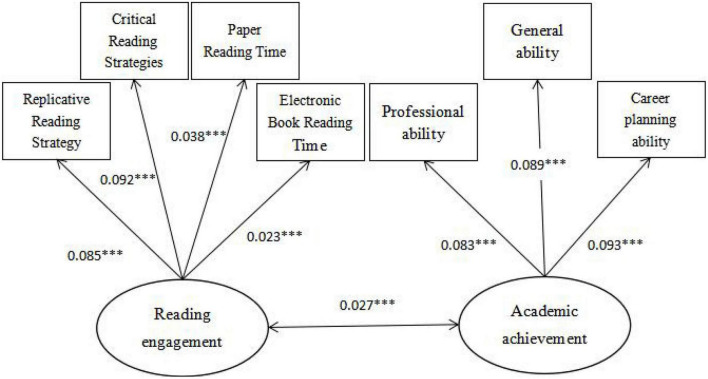
Construct validity analysis diagram of reading input (after standardization). Significance level *P < 0.1, **P < 0.05, and ***P < 0.01.

The paper used structural equation modeling to test the construct validity of the main variables, including academic achievement and reading engagement. As can be seen from Figure 1, the path coefficients of the three abilities of academic achievement are all above 0.7, and other observation indicators meet the requirements, indicating that the validity of the academic achievement in the questionnaire is good. As can be seen from Figure 2, the path coefficients of the two aspects of reading engagement are both above 0.6, and the other observed indicators are consistent, indicating that the validity of reading engagement in the questionnaire is good.

### Analysis of influencing factors of college students’ reading engagement

The above analysis shows that different individual characteristics, different reading motivation, different family reading culture, different reading habit cultivation and different organizational endowment of colleges and universities show different reading input. Therefore, the paper took college students’ reading engagement as the dependent variable and college students’ individual characteristics, reading motivation, family reading culture, college reading habit cultivation and college organizational endowment as independent variables to construct a multiple regression model and analyze the influencing mechanism of these factors on college students’ reading engagement. The regression results are shown in Table 5.

**TABLE 5:** Results of multiple linear regression of college students’ reading engagement.

		Reading engagement			
		Paper reading time	Electronic reading time	Replicative reading strategy	Critical reading strategies
Personal feature	Gender	0.007	0.001	0.005	−0.055**
	Grade	0.099***	0.058*	0.052**	0.013
	Discipline	–0.018	–0.047	0.028	0.017
	Origin of students	0.014	–0.018	0.013	–0.016
Reading motivation	Motivation for practicality	0.123***	0.048	0.237***	0.248***
	Motivation for entertainment	0.100**	0.182***	0.096***	0.137***
	Motivation of development	0.010	−0.087**	0.191***	0.280***
Family reading culture	Father’s years of education	−0.080*	–0.064	–0.052	–0.037
	Mother’s years of education	0.071	–0.010	0.044	0.015
	Family book collection	0.088**	0.120***	0.029	0.064**
	Family reading education	0.072**	0.071**	0.167***	0.134***
	Family reading atmosphere	0.173***	0.123***	0.066**	0.052**
Reading habits cultivation in universities	Course driven mechanism	0.058*	0.077***	0.070**	0.065**
	Resource Support Mechanism	–0.04	–0.033	0.037	0.048*
	Atmosphere excitation mechanism	0.118**	0.070*	0.089**	0.092**
	Interactive facilitation mechanism	–0.019	–0.93	0.03	0.014
Organizational endowment of colleges and universities	Types of universities	0.112***	0.108**	0.131***	0.075**
R squared after adjustment		16.90%	12.70%	39.30%	51.50%

Significance level *P < 0.1, **P < 0.05, ***P < 0.01.

#### The influence of college students’ individual characteristics on reading engagement

As can be seen from Table 5, in the prediction of paper reading time, the grade of college students has a positive and significant effect on reading time, that is, with the rise of grade, college students will spend more and more time on paper reading. In the prediction of reading strategies, the grade of college students plays a significant role in the replication reading strategy. With the rise of grade, college students are more and more inclined to choose the replication reading strategy. Gender of college students plays a significant role in critical reading strategies, and male students are more inclined to think and reflect when reading.

#### The influence of college students’ reading motivation on reading engagement

As can be seen from Table 5, in terms of reading motivation, both practical motivation and entertainment motivation have positive and significant effects on paper reading time, which also indicates that college students spend more time on practical and entertainment paper reading materials.

In terms of reading motivation, entertainment motivation has a positive and significant effect on e-reading time, while developmental motivation has a negative and significant effect on e-reading time. This also indicates that the main purpose of e-reading is entertainment, but college students choose to read from the perspective of their own development, so they spend less time on e-reading.

In terms of the impact of reading motivation on reading strategies, practical motivation, entertainment motivation and developmental motivation all play a positive and significant role in the replication and reflective reading strategies. In other words, on the whole, the stronger the reading motivation of college students, the better they will use the replication and reflective reading strategies.

#### The influence of college students’ family reading culture on reading engagement

As can be seen from Table 5, in the prediction of reading time, only the father’s education years play a negative and significant role in the paper reading time of college students. That is, the higher the father’s education level, the less reading time of college students, which is obviously not in line with our conventional cognition, and the specific reasons need to be further analyzed.

However, the amount of family books, family reading education and family reading atmosphere all have significant positive effects on the paper reading time and electronic reading time of college students, which indicates that the more books in the family, the better the family reading education, the better the family reading atmosphere, and the longer the reading time of college students.

In the prediction of reading strategies, family reading education and family reading atmosphere had positive and significant effects on college students’ duplicative reading strategies and reflective reading strategies. The amount of family books has a positive and significant effect on the reflective reading strategy, which also indicates that family reading education, family reading atmosphere and the amount of family books are an important factor affecting the reading strategy of college students.

#### Influence of reading habit cultivation on reading input in colleges and universities

In terms of the prediction of reading time, the course-driven mechanism and the atmosphere stimulation mechanism have positive and significant effects on paper reading time and electronic reading time, which indicates that the opening of college guidance courses and the development of college reading activities will increase the reading time of college students.

In the prediction of reading strategies, the course-driven mechanism and the atmosphere stimulating mechanism play a positive and significant role in the replication reading strategy of college students, while the course-driven mechanism, the resource supporting mechanism and the atmosphere stimulating mechanism play a positive and significant role in the reflective reading strategy of college students. Generally speaking, the cultivation mechanism of college reading habits plays a very important role in the selection of reading strategies for college students. The more attention colleges pay to the cultivation of students’ reading habits, the better college students can use reading strategies.

#### Influence of organizational endowment on reading input in colleges and universities

In the prediction of organizational endowment of colleges and universities, the type of colleges and universities plays a positive and significant role in paper reading time, electronic reading time, replicative reading strategy and reflective reading strategy, that is, with the improvement of college selection, the longer the reading time of college students and the better the use of reading strategies.

### Analysis of the impact of college students’ reading engagement on college students’ academic achievement

Through the above analysis, we can find that college students with different levels of reading engagement show different levels of academic achievement. Therefore, taking college students’ reading engagement as the independent variable, college students’ academic achievement as the dependent variable, and college students’ individual characteristics, reading motivation, family reading culture and organizational endowment as control variables, we constructed a multiple regression model to test whether college students’ reading engagement has an impact on college students’ academic achievement. At the same time, it also examines whether the individual characteristics of college students, reading motivation, family reading culture and organizational endowment of colleges and universities affect the academic achievement of college students. The regression results are shown in Table 6.

**TABLE 6:** Results of multiple linear regression of college students’ academic achievement.

Explanatory variable			Academic achievement of college students		
			Specialty literacy	General ability	Job and career planning
College students’ reading engagement	Reading time	Paper book reading time	0.092***	0.013	0.038
		Electronic book reading time	–0.007	–0.012	–0.023
	Reading strategies	Replicative reading strategy	0.054**	0.023	0.037
		Critical reading strategies	0.099**	0.136**	0.121**
Personal feature	Gender		0.020	–0.002	0.024
	Grade		–0.012	−0.058**	−0.051*
	Discipline		–0.033	−0.065**	−0.061**
	Origin of students		0.056**	0.055**	0.041
Reading motivation	Motivation for practicality		0.211	0.109***	0.187***
	Motivation for entertainment		–0.034	0.056*	0.012
	Motivation of development		0.243***	0.233***	0.26***
Family reading culture	Father’s years of education		−0.093**	–0.022	–0.032
	Mother’s years of education		0.022	–0.021	–0.015
	Family book collection		0.027	0.012	0.005
	Family reading education		–0.077	0.045	0.021
	Family reading atmosphere		–0.006	–0.014	–0.024
Organizational endowment of universities	Types of universities		0.009	0.009	0.081**
R squared after adjustment			58.30%	48.30%	62.70%

Significance level *P < 0.1, **P < 0.05, ***P < 0.01.

#### The influence of college students’ reading engagement on their academic achievement

(1) The influence of college students’ reading engagement on college students’ professional quality.

As can be seen from Table 6, paper reading time, replicative reading strategy and speculative reading strategy all play a significant positive role in the prediction of professional literacy of college students. However, e-reading time has no significant effect on college students’ professional quality. The explanatory power of the whole model is 58.3%. The data show that paper reading time and critical reading strategy can promote the improvement of college students’ professional ability, that is, the more paper reading time of college students, the more professional quality can be promoted. The use of critical reading strategy can also significantly improve the professional quality of college students. The measurement of professional literacy includes the improvement of professional performance and self-directed learning ability. It can be seen that if college students want to improve their professional knowledge and enhance their autonomous learning ability, they must first increase their reading time of paper books. Secondly, we should use the critical reading strategy, that is, we should keep thinking while reading, so as to improve our professional ability.

(2) The influence of college students’ reading engagement on college students’ general ability.

As can be seen from Table 6, only the critical reading strategy plays a significant positive role in predicting the general ability of college students. Paper reading time, electronic reading time and Replicative reading strategy have no significant effect on college students’ general ability. The explanatory power of the whole model is 48.3%. The data show that the critical reading strategy can promote the general ability of college students. The measurement of general ability includes two elements: expressive ability and organizational leadership ability. From the perspective of the function of reading, if college students want to improve their general ability, that is, their expressive ability and organizational leadership ability, they must strengthen the use of critical reading strategies in reading, think in reading, and constantly improve their general ability.

(3) The impact of reading engagement on college students’ career and career planning

As can be seen from Table 6, in terms of the prediction of career and career planning ability, the critical reading strategy has a significant positive effect on college students’ career and career planning ability. Paper reading time, electronic reading time and replicative reading strategy have no significant effect on college students’ general ability. The explanatory power of the whole model is 62.7%. The data show that the critical reading strategy can improve college students’ career and career planning ability. The measurement of career and career planning ability includes two elements: determining future career direction and forming stage development goals. The more college students use critical reading strategies in reading, the more they can improve their career and career planning ability.

#### Path analysis of the impact of college students’ reading engagement on academic achievement

The above multiple linear regression analysis shows the degree of influence of college students’ reading engagement on academic achievement. For how each dimension of college students’ reading engagement affects college students’ academic achievement, it is necessary to construct structural equation model to conduct path analysis and analyze the specific impact mechanism of college students’ reading engagement on academic achievement. AMOSS20.0 was used for path analysis in this paper. The chi-square of the model was 135.873, the degree of freedom was 13, and the overall significance probability of the model was P&LT. 0.001, the model fit is good, and the indicators are shown in Table 7.

**TABLE 7:** Fitting index of the model.

RMSEA	GFI	AGFI	NFI	CFI	IFI	TLI	RFI	Chi-square value	df	*P*
0.091	0.968	0.931	0.968	0.971	0.971	0.953	0.948	135.873	13.000	0.000

**FIGURE 3:**
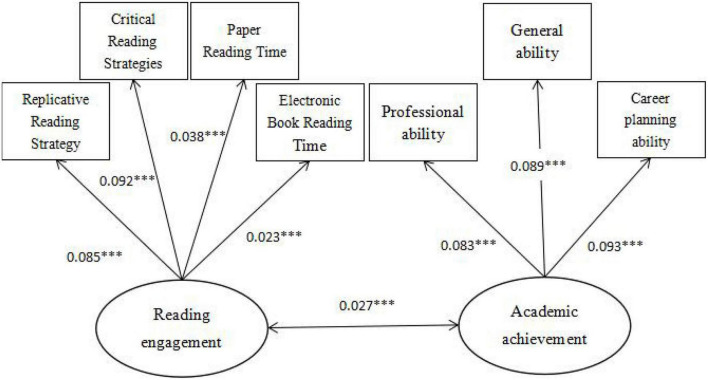
Structural equation model of college students’ reading engagement and academic achievement (after normalization). Significance level *P < 0.1, **P < 0.05, and ***P < 0.01.

According to the Maximum Likelihood method, the model is analyzed. Figure 3 summarizes the standardized influence mechanism between “reading engagement and academic achievement.” The effect of college students’ reading engagement on standardized academic achievement reached 0.57, and the probability of significance was P&LT. 0.001, which indicates that college students’ reading engagement can indeed have a positive role in promoting academic achievement. This also confirms the conclusion in the regression model that college students’ reading engagement has a significant promoting effect on academic achievement, and verifies the basic hypothesis of this paper.

#### The impact of other factors on college students’ academic achievement

In this study, the individual characteristics, reading motivation, family reading culture of college students and the organizational endowment of colleges and universities were added into the regression model as control variables. The regression model was used to test whether college students’ individual characteristics, reading motivation, family reading culture, reading habit training in colleges and universities, and organizational endowment in colleges and universities would have an impact on college students’ academic achievement. The regression results are shown in Table 6.

(1) The influence of college students’ individual characteristics on college students’ academic achievement.

In terms of professional literacy, it can be seen from Table 6 that both the place of origin and developmental motivation have significant positive effects on college students’ professional literacy, that is, the professional literacy of urban students is better than that of rural students, and the more college students read for their own development, the stronger their professional literacy will be.

In terms of general ability, it can be seen from Table 6 that grade, subject and place of origin all have negative and significant effects on college students’ general ability, that is, with the improvement of college students’ grade, their own general ability decreases. Compared with students majoring in science and engineering, students majoring in humanities and social sciences improve their general ability better. The origin of students has a positive and significant effect on the general ability of college students. Urban students improve their general ability more than rural students.

In terms of college students’ career planning ability, from Table 6, the grade of career and career planning ability of college students has negative significant effect, namely as college students’ grade rises, the cognitive ability of college students decreases in their career planning, and the reason may be that as their grade grows, especially when they need to find a job, their self-efficacy for their career planning ability becomes even lower. Discipline has a significant negative effect on career and lifetime planning ability, that is, the improvement of career planning ability of humanities and social science students is higher than that of science and engineering students.

(2) The impact of reading motivation on the academic achievement of college students.

In terms of professional quality, Table 6 shows that developmental motivation has a significant positive effect on the professional quality of college students, which indicates that the more they pay attention to long-term development in reading, the higher their career planning ability will be.

In terms of general ability, Table 6 shows that practical motivation, recreational motivation and developmental motivation all have a significant positive effect on general ability, which indicates that the stronger the reading motivation of college students, the better they can improve their general ability.

In terms of career planning ability, Table 6 shows that practical motivation and developmental motivation have a significant positive effect on career planning ability, which shows that the more college students pay attention to practicality and long-term development in reading, the better they can improve their career planning ability.

(3) The influence of family reading culture on the academic achievement of college students.

In terms of professional quality, Table 6 shows that the schooling years of fathers have a significant negative effect on the professional quality of college students, which is contradictory to conventional understanding. The possible reason is that most of the students come from private colleges and are not enthusiastic about their major. So the more educated their father, the more they will be motivated to learn knowledge. That being said, due to their psychological inversion, their professional quality is usually not very good.

In terms of general ability and career planning ability, Table 6 shows that the variables of family reading culture have no significant impact on the dimensions of college students’ academic achievement.

#### The impact of the organizational endowment of colleges on the academic achievement of college students

In terms of the organizational endowment of colleges, that is, the types of colleges, Table 6 shows that the types of colleges have a significant positive impact on students’ general ability, and as college selection improves, college students can better improve their general ability. This shows that the better the organizational endowment of colleges, the more prominent the general abilities of college students, including organizational and communication skills.

## Conclusion and recommendation

### Conclusion

#### Factors affecting college students’ reading engagement

(1) From the perspective of college students, in terms of college students’ reading time, grades, practical motivation, recreational motivation, family collection of books, family reading education, and family reading atmosphere have a significant positive impact on paper reading time. The same is true of the education level of fathers. College students’ grade, recreational motivation, developmental motivation, family book collection, family reading education, and family reading atmosphere have a significant positive impact on e-reading time. In terms of reading strategies, grades, practical motivation, recreational motivation, developmental motivation, family reading education, and family reading atmosphere have significant positive effects on replicative reading strategies, while gender, practical motivation, recreational motivation, developmental motivation, family book collection, family reading education, and family reading atmosphere have significant positive effects on critical reading strategies.

(2) From the perspective of colleges, the curriculum-driven mechanism, atmosphere stimulation mechanism, and organizational endowment have a significant positive impact on paper reading time and e-reading time. In terms of reading strategies, curriculum-driven mechanism, atmosphere-based incentive mechanism, and college organizational endowment have a significant positive impact on replicative reading, while curriculum-driven mechanism, resource support mechanism, atmosphere incentive mechanism, and college organizational endowment have a significant positive impact on critical reading.

#### Mechanism of factors affecting the academic achievement of college students

From the analysis on how college students’ reading engagement affect their academic achievement, the following two points are drawn: (1) Among others, reading time is the most important factor affecting reading behavior and reading volume. It is found that the reading time of paper books has a significant positive effect on the professional quality of college students, but has no significant effect on the general ability, career and lifetime planning ability of college students. Many students prefer paper books while studying professional knowledge, because they can take notes on them and they are conducive to systemic learning. This may be an important reason why paper-book reading time has a significant positive effect on the professional quality of college students. In addition, e-book reading time has no significant effect on the three abilities concerning college students’ academic achievement, which to some extent reflects the drawbacks of e-book reading. E-book reading can easily lead to fragmented reading, and it is difficult to form a systematic knowledge system. Moreover, students also reported that e-books are basically recreational. Therefore, it is difficult for such reading to have a significant impact on the three abilities, but this does not mean that e-reading is useless. (2) In terms of reading strategies, critical reading strategies have a significant positive effect on college students’ professional quality, general ability, career and lifetime planning. While reading, college students should not only rely on passive memory, but also learn to think and make explorations actively, which is conducive to the improvement of academic achievement.

Based on the analysis on how the individual characteristics, reading motivation, family reading culture and institutional endowment of college students on the academic achievement of college students, the following four conclusions are drawn: (1) Grades have a significant negative effect on college students’ general ability and career and lifetime planning ability. With the increase of grades, the general ability and the ability of career and lifetime planning decrease. Disciplines have a significant negative effect on the general ability and ability of career and lifetime planning: feedback from students of humanities and social sciences shows that they can better improve their general ability through reading than those of science and engineering. The source of students has a positive effect on the professional quality, general ability, career and lifetime planning of college students, that is, the self-evaluation of urban students on their academic achievement is higher than that of rural students. (2) Practical motivation has a positive and significant effect on college students’ general ability and career planning ability. Students believe that practical reading can promote the two abilities; recreational motivation has a significant impact on general ability; motivation has a significant positive effect on academic achievement overall. (3) In terms of family reading culture, only the education level of fathers has a significant negative effect on college students’ professional quality, while that of others has no significant effect, which is contrary to our cognition and requires further verification and analysis. (4) The organizational endowment of colleges has a significant positive effect on general ability. With the improvement of college selectivity, college students have a higher evaluation of their general ability.

### Recommendation

This research verifies the functional mechanism of reading subject, reading object and reading carrier, and concludes that the core of the operating mechanism of college reading management lies in the management of reading input (reading time and reading strategy), and the mechanism of college reading management makes sure that the reading subject, reading object and the reading carrier are effectively integrated. The joint effect of the operation mechanism and the action mechanism of reading management in colleges can promote the effective implementation of reading management, and then improve the academic achievement of college students.

First of all, find the individual, group and epochal characteristics of college students, and based on systematically grasping their individual characteristics, encourage them to read and improve their reading efficiency. Based on the cultivation of reading content tendencies and reading habits, actively promote the application and conversion of replicative reading and critical reading. Second, give play to the role of the reading object, well manage and serve knowledge content, promote the transformation between tacit knowledge and explicit knowledge, and play the role of tacit knowledge and tacit curriculum. In particular, promote reading atmosphere and provide a comfortable reading space for students to combine the role of the environment and culture in talent cultivation. Finally, in terms of reading carriers, combine technology and reading form. The construction of resources and platforms in colleges is the foundation of reading management. The construction and application of electronic resources and online platforms has become an inevitable trend under the background of technological change. The application of new technologies to improve and promote reading, especially the method of “intelligence plus reading” will be the main trend leading the development of reading carrier management.

#### Improve the construction of reading system

A sound system can guarantee the effective operation of reading management. The study found that the intended purpose of reading management cannot be achieved only through encouragement and advocacy. It is also necessary to systematically build a flexible and rigid institutional system that combine universities, libraries, counselors, teachers and student.

(1) In the construction of flexible restraint system, requirements concerning the number of books that students should read (for example one hundreds Chinese and foreign classics) and thought sharing should be implemented in each term, and relevant achievements, such as the number of book sharing and essays on book reading, are directly linked to student awards, party membership, and student cadre elections.

(2) In terms of rigid system construction, the responsibility assessment system for university administrators such as librarians, counselors and teachers should consider whether they participate in and guide students’ reading activities. Libraries and curators promote reading services and counseling, extend the functions of libraries in a timely manner based on technological progress and actual needs. In particular, they are responsible for selecting and recommending classic books and offer monthly lectures on famous classics. The work assessment system for counselors should consider whether they organize and take part in reading group activities in class management activities. Teachers, especially those with senior professional titles, should regularly offer lectures on professional reading, which has become an important part of teacher assessment. At the same time, colleges have established a reading assistance system for students with financial difficulties through student scholarships and other means.

#### Enrich reading resources and promote platform building

Reading resources and platforms are the material basis of reading management in colleges. Efforts should be made to strengthen the information construction of libraries, encourage teachers to develop relevant reading websites, share proper reading resources among students, and students with a “reading corner” for sharing and reading, thus building a bridge connecting teachers and students and promoting communication among students. In addition, new technologies should be used to improve college students’ reading strategies. In particular, the method of “artificial intelligence plus reading” such as virtual reality and augmented reality can effectively resolve shortcomings caused by “fragmented reading,” and find a reasonable and effective way for the promotion of classic reading.

#### Build a better long-term reading mechanism

(1) Establish a mechanism for reading promotion. The formation of a reading culture is a long-term and accumulative process, which cannot be achieved by just one or two reading activities. Through online and offline reading tutoring and various reading promotion activities (special lectures, reading salons, etc.), colleges can effectively improve students’ reading enthusiasm and participation, and form an institutionalized and systematic reading tradition. Libraries should improve their reading service, and introduce themselves to freshmen so that they know their services. Efforts should also be made to optimize the information building, and build a recommendation column for new and good books. At the same time, we should focus on guiding students to use electronic resources, and conduct regular activities to let them know how to use electronic resources better.

(2) Build a reading interaction system. The reading interaction between teachers and students and among students is an important part of campus interaction, and also a beautiful “learning landscape” on campus. On the one hand, it is necessary to construct a benign reading interaction mechanism between teachers and students, involving the reading interaction between professional teachers and students, and between counselors and students, and the informal reading organization of teachers and students. On the other hand, it is also necessary to build an active classmate reading interaction mechanism that include formal reading interaction and informal reading activities.

(3) Build a development mechanism for college students’ reading behavior. First, increase the reading time. Through opening characteristic reading courses, building a wealth of electronic reading resources, and providing an elegant and comfortable reading environment on campus, students are encouraged to read at any time. Second, use appropriate reading strategies. Encourage teachers to offer guidance on reading in their teaching process; organize clubs and library activities; invite famous teachers to give reading strategy lectures. Third, optimize the structure of reading contents, help students choose reading contents reasonably based on their needs and major, promote classic reading, and prevent recreational reading from becoming the main body of reading.

### Research outlook

(1) Expand the distribution area and number of samples. The follow-up research will cover more samples, select some colleges and universities in the eastern, central and western regions as the survey objects, expand the sample size to about 4,000, and conduct interviews with graduates and teachers to further explore factors influencing college students’ reading and their relationship with academic achievement.

(2) Systematically carry out reading management in colleges and universities. Reading management needs to implement targeted reading intervention measures for 2–3 years and quantify specific indicators, which will inevitably produce good practical results. Reading management in colleges and universities should further expand its thinking and pay attention to the reading intervention of college students before they go to college. Especially for students who come from rural areas and have a relatively weak cultural background, it is necessary to transfer reading management to the stage of compulsory education.

(3) Promote classic reading. Classical reading has always been the most important part among college students, but there are various reading difficulties in practice. How to use the reform of reading carriers brought by technological reform, especially the method of “artificial intelligence plus classics” in practice, to improve the popularity and efficiency of classic reading is one of focuses for follow-up research.

(4) Research on the relationship between e-reading and academic achievement. With the increase of electronic resources and the popularization of media such as film, television media and e-readers, television, the Internet, and mobile phones have become the major reading methods used by the Chinese people. How to effectively use the “artificial intelligence plus reading” method, especially the application of VR and AR, to improve the academic achievement of college students, is worthy of further research in the context of technological innovation.

## Data availability statement

The raw data supporting the conclusions of this article will be made available by the authors, without undue reservation.

## Author contributions

X-WW proposed the research hypothesis and research design, analyzed the experimental data, and analyzed the experimental results. Y-JZ contributed to questionnaires and collected data. Y-CZ designed the framework of the manuscript and discussed the experimental results. All authors contributed to the article and approved the submitted version.
